# Primary tumour–vessel tumour–nodal tumour classification for patients with invasive ductal carcinoma of the breast

**DOI:** 10.1038/sj.bjc.6602353

**Published:** 2005-03-01

**Authors:** T Hasebe, S Sasaki, S Imoto, N Wada, G Ishii, A Ochiai

**Affiliations:** 1 Division of Pathology, The National Cancer Center Research Institute East, Kashiwanoha 6-5-1, Kashiwa, Chiba 277-8577, Japan; 2 Division of Epidemiology, The National Cancer Center Institute East, Chiba, MD, Japan; 3 Department of Breast Surgery, The National Cancer Center Hospital East, Chiba, MD, Japan

**Keywords:** invasive ductal carcinoma, prognosis, histology, pTNM, Nottingham Prognostic Index, classification

## Abstract

There are many studies that show biological differences between invasive ductal carcinoma (IDC) with and without nodal metastasis, but no prognostic classification taking into consideration any biological differences between them is currently available. We previously investigated the histological characteristics that play an important role in tumour progression of IDCs according to their nodal status, and a new prognostic histological classification, the primary tumour–vessel tumour–nodal tumour (PVN) classification, was devised based on the histological characteristics of IDCs with and without nodal metastasis. Multivariate analyses using the Cox proportional hazard regression models were used to compare the ability of the PVN classification to predict tumour recurrence and death in 393 IDC patients based on the following histological classifications: (1) the pTNM classification, (2) the Nottingham Prognostic Index, (3) the modified Nottingham Prognostic Index, and (4) the histologic grade. In IDCs without nodal metastasis, only the PVN classification significantly increased the hazard rates (HRs) of tumour recurrence and death (*P*<0.05), independent of the hormone receptor status. Similarly, in IDCs with nodal metastases, only the PVN classification significantly increased the HRs of tumour recurrence and death (*P*<0.05), independent of the hormone receptor status. We conclude that the PVN prognostic histological classification is the best classification available for IDC of the breast.

There are many studies that show differences between the prognostic parameters of the primary-invasive tumours of invasive ductal carcinoma (IDC) patients with and without nodal metastasis (Gilchrist *et al*, 1993; Pedersen *et al*, 2000; Depowski *et al*, 2001; Scorilas *et al*, 2001), and the former show a significantly worse prognostic course than the latter. Thus, it is necessary to accurately predict the difference in the malignant potential of IDCs with and without nodal metastasis from the viewpoint of neoadjuvant or adjuvant chemotherapy for IDC patients.

We recently clearly demonstrated that the histological characteristics of tumour cells in lymph vessels, blood vessels, and lymph nodes play a very important role in tumour progression of IDCs of the breast (Hasebe *et al*, 2002b, 2003a, 2003b, 2004a). Among these histological characteristics, we clearly identified the most important histological characteristics predicting the outcome of IDC patients with and without nodal metastasis (Hasebe *et al*, 2004b). Our studies clearly showed that the prognostic histological characteristics of primary-invasive tumours and tumour cells in vessels differ in IDCs without and with nodal metastasis, and that the histological characteristics of tumours in lymph nodes play a more important role in the outcomes of IDC patients with nodal metastasis than those of the primary-invasive tumours or tumour cells in vessels. These findings strongly suggest the characteristics of IDCs with and without nodal metastasis are different. We therefore attempted to establish separate new histological prognostic classifications consisting of histological characteristics suitable for IDC patients with and without nodal metastasis based on a total evaluation of the histological characteristics of the primary-invasive tumours and tumour cells in vessels and lymph nodes.

The pTNM classification, the Nottingham Prognostic Index (NPI), and histologic grade are the major histological prognostic classifications currently used clinically to predict the outcome of patients with IDC (Bloom and Richardson, 1957; Todd *et al*, 1987; Elston and Ellis, 1991; Sundquist *et al*, 1999; Sobin and Witterkind, 2002). Nevertheless, these prognostic classifications use histological characteristics common to both primary-invasive IDC tumours with and without nodal metastasis, meaning that they do not take into consideration any differences between IDCs with and without nodal metastasis.

The purpose of this study was to establish separate prognostic histological classifications for IDC patients with and without nodal metastasis based on not only the histological characteristics of the primary-invasive tumours but also of the tumour cells in lymph vessels, blood vessels, and nodal metastatic tumours according to hormone receptor status. The results clearly demonstrated that the newly proposed prognostic histological classifications are the best classifications available for IDC of the breast.

## MATERIALS AND METHODS

### Patients

A total of 392 consecutive cases of IDC of the breast surgically treated between July 1992 and November 1998 at the National Cancer Center Hospital East served as the basis of this study. Clinical information was obtained from the patients' medical records after complete histological examination of all of the IDCs. All patients were Japanese women, and they ranged in age from 28 to 78 years (mean, 51 years). All had a solitary lesion. In total, 209 patients were premenopausal, and 183 were postmenopausal. Partial mastectomy was performed in 55, modified radical mastectomy in 313, and standard radical mastectomy in 24 patients. Axillary lymph node dissection consisting of levels I, II, ±III was carried out in all patients. None of the patients had received radiotherapy or chemotherapy before surgery. Adjuvant therapy was performed in 289 patients. Of the 188 IDC patients without nodal metastasis, 88 received no adjuvant therapy, 24 received tamoxifen, 45 received CMF (cyclophosphamide, methotrexate, and 5-FU), AC (adriamycin and cyclophosphamide), or EC (epirubicin and cyclophosphamide), and 31 received chemotherapy plus tamoxifen. Of the 204 IDC patients with nodal metastases, 14 received no adjuvant therapy, 34 received tamoxifen, 51 received chemotherapy, and 105 received chemotherapy plus tamoxifen. There were no cases of inflammatory breast cancer in this series. Oestrogen receptors (ERs) and progesterone receptors (PRs) in the cytosol fractions were determined by enzyme immunoassay (Otsuka Assay Laboratory, Tokushima, Japan). The upper cutoff values of the ER assay and PR assay were 13 fmol mg^−1^ protein and 10 fmol mg^−1^ protein, respectively.

For pathological examination, the surgically resected specimens were fixed in 10% formalin, and multiple histological sections were taken from each tumour for histological examination without knowledge of the patient's outcome. The sections were processed routinely and embedded in paraffin.

### Proposed new prognostic classifications

We attempted to establish new separate histological prognostic classifications, called the PVN (primary tumour–vessel tumour–nodal tumour) classifications, for IDC patients with and without nodal metastasis. The PVN classifications for IDCs with and without nodal metastases were devised based on the histological characteristics of the tumour that were found to be most important in predicting the outcome of IDC patients in the previous studies (Hasebe *et al*, 2004b). The parameters of the PVN classification for IDCs with and without nodal metastasis are listed in Tables 1 and 2. The method for assessing each parameter is described in the previous studies (Hasebe *et al*, 2002b, 2003a, 2003b, 2004a, 2004b). In the PVN classification for IDC without nodal metastasis (Table 1), a score of 1 point each was given for an FF >8 mm in dimension (Hasebe *et al*, 2000, 2002a), lymph vessel tumour emboli with >6 apoptotic figures, lymph vessel tumour emboli >3 mm distant from the primary-invasive tumour margin, and >2 blood vessels invaded. In the classification of IDC with nodal metastasis (Table 2), a score of 1 point each was given for tumour necrosis (Gilchrist *et al*, 1993), severe nuclear atypia of the primary-invasive tumours, >5 lymph vessels invaded, >2 apoptotic figures in blood vessel tumour emboli, >5 lymph nodes with extranodal invasion, and >5 mitotic figures in nodal metastatic tumours. A score of 0 was given for each of the above that was absent. The total PVN scores for IDCs with and without nodal metastasis were calculated.

### Prognostic histological classifications for comparative study

The following existing histological classifications were compared with our proposed new classification in regard to prediction of disease-free and overall survival: (1) pTNM (Sobin and Witterkind, 2002), (2) NPI (Todd *et al*, 1987; Sundquist *et al*, 1999), and (3) HG (Bloom and Richardson, 1957; Elston and Ellis, 1991). The NPI classification generally consisted of the following three groups: low, NPI ⩽3.4; intermediate, 3.4<NPI⩽5.4; high, NPI>5.4. However, we additionally divided the intermediate NPI group into an intermediate low group (3.4<NPI⩽4.4), and an intermediate high group (4.4<NPI⩽5.4), and this classification was regarded as a modified NPI and compared with the PVN classification to predict the outcome of the IDC patients.

### Outcome

Patient survival was evaluated by follow-up for a median period of 94 months, ranging in months from 61 to 136 months as of November 2003. A total of 106 patients experienced tumour recurrence, and 83 had died of their disease. Disease-free and overall survival were measured from the date of surgery. Metastasis or local recurrence was considered evidence of tumour relapse. Only deaths due to breast cancer were considered for the purposes of this study.

### Statistical analysis

We divided each classification into four groups (IDCs without nodal metastasis: low (score 0), intermediate (score 1), high (score 2), and very high-risk groups (scores 3 and 4) (Table 1); IDCs with nodal metastasis: low (score 0), intermediate (scores 1 and 2), high (score 3), and very high-risk groups (score 4–6) (Table 2), based on significant associations with tumour recurrence or death in the univariate analyses by the Cox proportional hazard regression model (Cox, 1972). Survival curves were drawn following the Kaplan–Meier method (Kaplan and Meier, 1958). In IDCs without nodal metastasis, since the number of tumour recurrences or deaths was relatively small, when compared according to the hormone receptor status, the trend values of the hazard rate (HR), 95% confidence interval (CI), and the *P*-values for disease-free or overall survival were evaluated using a multivariate analysis with the Cox proportional hazard regression model. Since only five patients with IDCs without nodal metastasis and either or both positive for ER and PR died, a multivariate analysis could not be performed for overall survival. In IDCs with nodal metastasis according to hormone receptor status, since tumour recurrence and/or death was not observed in the low-risk groups of the PVN, NPI, modified NPI, pTNM, or HG classifications, the low- and intermediate-risk groups were taken together as a referent category to assess the HRs of tumour recurrence or death in the multivariate analyses. The predictive power for disease-free and overall survivals of each classification was evaluated by multivariate analysis using the Cox proportional hazard regression model (Cox, 1972).

All analyses were performed with Statistica/Windows software (StatSoft, Tulsa, Okla).

## RESULTS

### Tumour recurrence and death rates of the PVN classification

**Figure 1:**
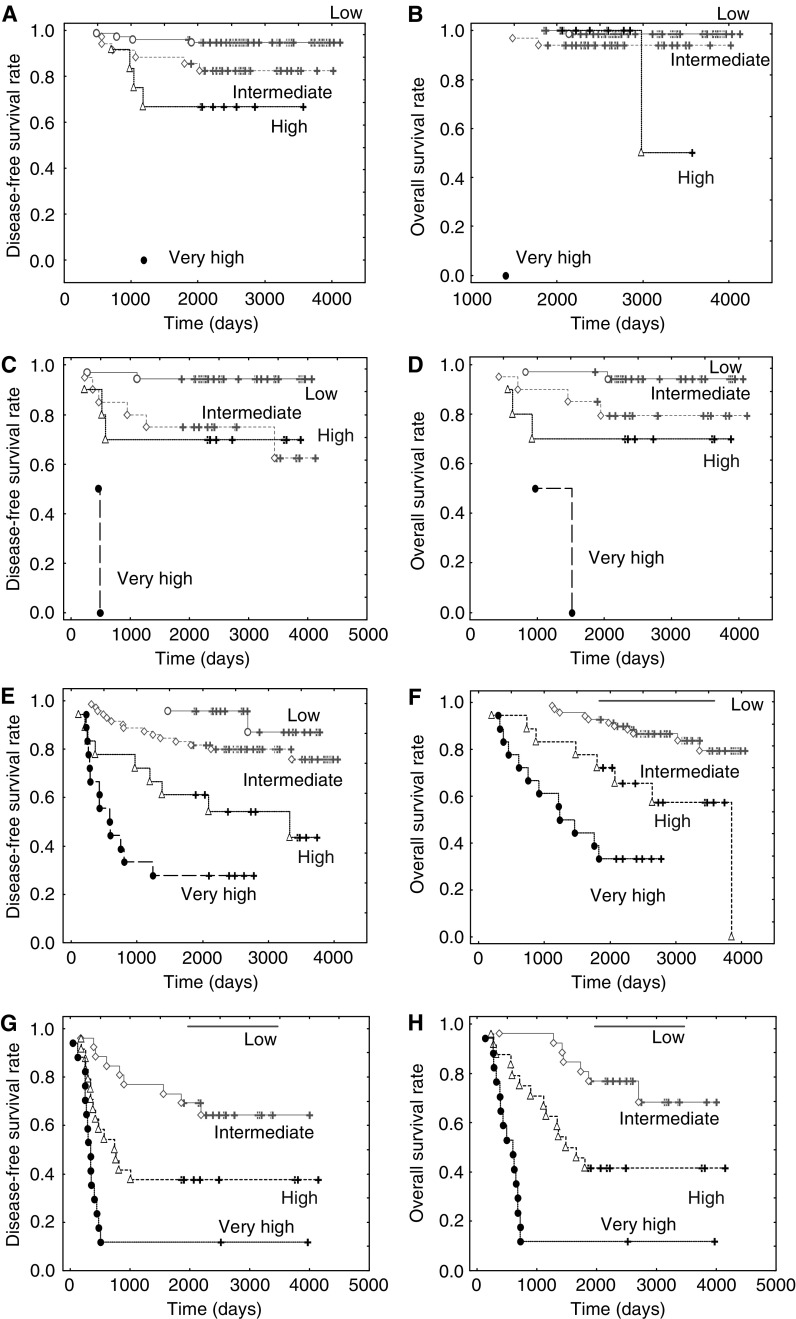
The disease-free and the overall survival curves according to the PVN classification system in IDCs without nodal metastasis that were positive for ER and/or PR (**A** and **B**), IDCs without nodal metastasis that were negative for both ER and PR (**C** and **D**), IDCs with nodal metastasis that were positive for ER and/or PR (**E** and **F**), and IDCs with nodal metastasis that were negative for both ER and PR (**G** and **H**). The disease-free and overall survival curves of each risk group in each IDC subgroups decrease according to the risk order of the classification, with the very high-risk groups in each IDC subgroup showing the shortest disease-free and overall survival curves independent of nodal status and hormone receptor status.

In IDCs without nodal metastasis, the largest numbers of patients were observed in the low-risk group, and the number of patients belonging to each group decreased in the risk order of the classification (Table 1). The rates of tumour recurrence or death of IDC patients increased in the score order of the classification, and all cases belonging to the very high-risk group showed tumour recurrence and death by 5 years after the initial operation. The PVN classification showed significantly short crude disease-free and overall survival periods in the risk order of the classification (*P*<0.001, Figure 1A–E).

In IDCs with nodal metastasis, the rates of tumour recurrence or death increased in the risk order of the classification, and the tumour recurrence of the low-risk group was observed only in one case, and no case died of the disease (Table 2). On the contrary, the tumour recurrence rate of the very high-risk group reached 80%, and the tumour death rate of the very high-risk group exceeded 70%. Crude disease-free and overall survival periods of the PVN classification were significantly shortened in the risk order of the classification (*P*<0.001, Figure 1E–H).

### Comparative studies of the PVN classification with pTNM, NPI, modified NPI, and HG classifications

In IDCs without nodal metastasis and positive for ER or PR or both, the PVN classification had the largest number of cases in the low-risk group, compared with all the prognostic classification systems studied, and the frequency of tumour recurrence or death in the low-risk group was similar to that of the other prognostic classifications (Table 3, Figure 1A and B). The frequency of tumour recurrence or death in the very high-risk group of the PVN classification was 100%. On the other hand, the frequencies of tumour recurrence or death in the high-risk groups of the other prognostic classification systems were very low. In the multivariate analyses, the PVN classification showed a significant trend for the HR, 95% CI, and the *P*-values for disease-free survival when compared with the other prognostic classification systems, and only the NPI classification also showed a significant trend for the HR, 95% CI, and the *P*-values for disease-free survival (Table 3).

In IDCs without nodal metastasis that were negative for both ER and PR, the PVN classification and the pTNM classification selected almost an equally large number of cases belonging to the low-risk group. However, the frequency of tumour recurrence or death in the very high-risk group of the former classification was 100%, while no cases of tumour recurrence or death were observed among the stage IIIB cases in the latter classification (Table 4, Figure 1C and D). The frequencies of tumour recurrence or death in the high-risk groups of the other prognostic classification systems were very low, and the NPI, modified NPI, and HG classifications could not narrow the number of cases belonging to each risk group in the risk orders of the classification systems. In the multivariate analyses, the PVN classification significantly increased the trend values for the HRs, 95% CI, and the *P*-values for disease-free and overall survival when compared with other prognostic classification systems (Table 4). On the other hand, other prognostic classification systems failed to significantly increase the trend values for the HRs, 95% CI, and the *P*-values when compared with the PVN classification.

In IDCs with nodal metastasis and positive for ER or PR or both, the rates of tumour recurrence or death increased according to the risk order of the PVN classification, and the rates of tumour recurrence and death in the very high-risk group were 72 and 62%, respectively (Table 5, Figure 1E and F). In the pTNM classification, the rates of tumour recurrence or death for cases classified as IIB, IIIA, or IIIB were similar, and the rates of tumour recurrence and death for IIIC cases were lower than those in the very high-risk group of the PVN classification. Although the rates of tumour recurrence or death in the NPI, modified NPI, and HG classifications increased according to the risk order of the classifications, the tumour recurrence and death rates in the high-risk groups were lower than those in the very high-risk group of the PVN classification. In addition, the NPI, modified NPI, and HG classifications could not narrow the number of cases belonging to each risk group in the risk orders of the classifications. In the multivariate analyses, the PVN classification significantly increased the HRs of tumour recurrence and death in the high and very high-risk groups when compared with the pTNM, NPI, and HG classifications (Table 5). On the other hand, only the pTNM classification significantly increased the HRs of tumour recurrence and death for the stage IIIC cases when compared with the PVN classification, and the NPI and HG classifications failed to increase the HRs of tumour recurrence or death when compared with the PVN classification. In a comparative study of the PVN classification and the modified NPI classification, although the PVN classification failed to significantly increase the HRs of tumour recurrence in the high risk-group, it significantly increased the HRs of tumour death in the high and very high-risk groups. On the other hand, the modified NPI classification showed a marginally significant increase in the HRs of tumour recurrence in the intermediate-high, and high-risk groups, but failed to significantly increase the HRs of tumour death in these groups.

In IDCs with nodal metastasis who were negative for both ER and PR, the rates of tumour recurrence or death according to the risk order of the PVN classification, and the rates of tumour recurrence and death in the very high-risk group were 88 and 88%, respectively (Table 6, Figure 1G and H). In the pTNM classification, the rates of tumour recurrence or death in the stage IIB, IIIA, or IIIB classifications were almost the same, and the rates of tumour recurrence and death in the stage IIIC classification were lower than those in the very high-risk group of the PVN classification. Although the rates of tumour recurrence or death in the NPI, modified NPI, or HG classifications increased according to the risk order of the classifications, the tumour recurrence and death rates in the high-risk groups of them were lower than those in the very high-risk group of the PVN classification. In addition, the pTNM, NPI, modified NPI, and HG classifications could not narrow the number of cases belonging to each risk group. Multivariate analyses showed that the high- and very high-risk groups of the PVN classification significantly increased the HRs of tumour recurrence and death when compared with the other prognostic classification systems (Table 6). On the contrary, no significant increases in the HRs of tumour recurrence or death were observed in the pTNM, NPI, modified NPI, and HG classifications in the multivariate analyses.

## DISCUSSION

The current study clearly demonstrated that the PVN classification is the only prognostic classification that can classify IDC patients into the four groups according to in the risk order of the classification with significant rates of tumour recurrence or death. In addition, only the PVN classification could select IDC patients with the very high risk of tumour recurrence or death independent of nodal status and hormone receptor status. Since the parameters of the PVN classification were selected based on the precise studies that evaluate the histological characteristics of the primary-invasive tumours, tumours in vessels, and those in lymph nodes (Hasebe *et al*, 2002b, 2003a, 2003b, 2004a, 2004b), they are most likely the most suitable ones to accurately assess the true biological malignant potential of IDCs. Therefore, we conclude that the PVN classification is the best prognostic histological classification available for IDCs of the breast.

The comparative studies also clearly demonstrated the merits and demerits of the other prognostic classifications in the prediction of the outcome of IDC patients. In IDCs without nodal metastasis, the pTNM and HG classifications could not significantly increase the trend values for the HRs of tumour recurrence or death in the multivariate analyses, but a significant increase in the trend values of the HRs of tumour recurrence was observed for the NPI classification in multivariate analyses with the PVN classification in IDCs positive for ER and/or PR. The pTNM classification evaluates the malignant potential of IDCs only according to the invasive tumour size of the primary tumours. The HG classification evaluates the degree of tubular formation, the degree of nuclear atypia, and the number of mitotic figures in tumour cells, but takes no account of invasive tumour size. The NPI classification evaluates the malignant potential of IDCs according to the HG and invasive tumour size of the primary invasive tumours. This strongly suggests that the NPI classification system contains more biological information based on the tumour histology of the primary invasive tumour cells than the pTNM classification and more biological information on the tumour size of the primary invasive tumours than the HG classification. Thus, the NPI classification can more precisely assess the malignant potential of IDCs positive for ER and/or PR than the pTNM and HG classifications, resulting in the superiority of the former to the latter in the prediction of the outcome of IDC patients without nodal metastasis who were positive for ER and/or PR. However, in IDCs negative for both ER and PR, the NPI classification failed to significantly increase the trend values of the HRs of tumour recurrence and death in the multivariate analyses with the PVN classification. Since IDCs that are negative for both ER and PR have a much higher malignant potential than IDCs that are positive for ER and/or PR, the NPI classification does not maintain its prognostic predictive power when compared with the PVN classification. In addition, this study clearly demonstrated that the modification of the NPI classification was of no benefit to accurate prediction of tumour recurrence or death in patients with IDCs without nodal metastasis.

In IDCs with nodal metastasis, the comparative studies with the PVN classification clearly demonstrated that the NPI, modified NPI, and HG classification were no use in the prediction of the outcome of IDC patients in the multivariate analyses, but in IDCs that are positive for ER and/or PR the pTNM classification showed significant trend *P*-values for tumour recurrence and death, and especially in stage IIIC cases a significant increase was seen in the HRs of tumour recurrence and death in the multivariate analyses. The HG classification ignores the nodal status of IDCs. The NPI and modified NPI classifications assess nodal status according to the number of nodal metastases: score 1, no nodal metastasis; score 2, one to three nodal metastases; and score 3, four or more nodal metastases. Although the pTNM classification also assesses nodal status according to the number of nodal metastases, the stage IIIC IDCs consist of IDCs with 10 or more nodal metastases, independent of their invasive tumour size. Thus, the assessment of 10 or more nodal metastases in the pTNM classification is probably very important for accurately predicting the outcome of patients with IDCs with nodal metastasis that are positive for ER and/or PR. The pTNM evaluations of stage IIB, IIIA, and IIIB cases failed to increase the HRs of tumour recurrence or death in the multivariate analyses, and these stages exhibited similar rates of tumour recurrence or death in IDCs with nodal metastases that are positive for ER and/or PR. In addition, the NPI and modified NPI node classifications did not improve the accurate prediction of tumour recurrence or death in patients with nodal metastasis who were positive for ER and/or PR. Therefore, the N1 and N2 categories of the pTNM classification probably have no effect on the accurate prediction of the outcome of IDC patients with nodal metastases. However, this study clearly demonstrated that in IDCs negative for both ER and PR, the stage IIIC of the pTNM classification failed to maintain its predictive prognostic power, when compared with the PVN classification. Based on these findings, the histological characteristics of the T and N categories of the pTNM classification should be improved, since the pTNM classification is the global prognostic classification for patients with IDC of the breast.

In conclusion, the current study clearly demonstrated that the PVN classification is by far the best histological classification for predicting the outcome of patients with IDC of the breast. Indeed, the methodology for determining the PVN classification may be more complex than those other existing classification systems, but the methods that evaluate the parameters of the PVN classification have been reported in our previous studies (Hasebe *et al*, 2002b, 2003a, 2003b, 2004a, 2004b), as well as other previous studies (Donegan *et al*, 1993; Gilchrist *et al*, 1993; De Jong *et al*, 2000). We confirmed the prognostic significance of the presence of tumour necrosis, the presence of apoptotic figures in the tumour cells, and the presence of extranodal invasion in our previous study (Hasebe *et al*, 2004b). In addition, Colpaert *et al* (2001) confirmed the prognostic significance of the presence of a fibrotic focus in node-negative IDCs. In the field of medical research, technology is developing daily, enabling many new important findings in the area of cancer research. Consequently, new methods should not only include primary tumour histology but also the histology of tumour in vessels and lymph nodes to accurately assess the true malignant potential of IDCs. Since the PVN classification provides pathologists with more precise information on the malignant potential of IDCs than other prognostic classification systems, pathologists should make efforts to assess the true malignant potential of IDCs using the criteria of the PVN classification.

## Figures and Tables

**Table 1:** PVN classification of IDC without nodal metastasis and tumour recurrence and death rates of PVN classifications at 5 years after the initial operation

**Parameter**	**Score**
(1) Fibrotic focus, dimension, in primary-invasive tumours	
Absent/⩽8 mm *vs* >8 mm	0 *vs* 1
(2) Number of apoptotic figures in lymph vessel tumour emboli	
Absent/⩽6 *vs* >6	0 *vs* 1
(3) Distance of lymph vessel tumour emboli from primary-invasive tumour margin	
Absent/⩽3 mm *vs* >3 mm	0 *vs* 1
(4) Number of blood vessel invasion	
Absent/⩽2 *vs* >2	0 *vs* 1
	Total: 0–4
	
Tumour recurrence and death rates according to the PVN scores at 5 years from the initial operation	

**Classes (scores)**	**Cases**	**TRR (%)**	**MR (%)**
Low (0)	109	5 (5)	1 (1)
Int (1)	54	10 (19)	5 (9)
High (2)	22	7 (32)	3 (14)
Very high (3/4)	3	3 (100)	3 (100)
Total	188	25	12

PVN=primary tumour–vessel tumour–nodal tumour; absent=absent for event; IDC=invasive ductal carcinoma; TRR=tumour recurrence rate; MR=mortality rate; Int=intermediate; 3/4=3 and 4.

**Table 2:** PVN classification of IDC with nodal metastases, and tumour recurrence and death rates of the PVN classification at 5 years after the initial operation

**Parameters**	**Score**
(1) Tumour necrosis in primary-invasive tumours	
Absent *vs* present	0 *vs* 1
(2) Nuclear atypia of primary-invasive tumours	
Mild/moderate *vs* severe	0 *vs* 1
(3) Number of lymph vessels invasion	
Absent/⩽5 *vs* >5	0 *vs* 1
(4) Number of apoptotic figures in blood vessel tumour emboli	
Absent/⩽2 *vs* >2	0 *vs* 1
(5) Number of lymph nodes with extranodal invasion	
No/⩽5 *vs* >5	0 *vs* 1
(6) Number of mitotic figures of nodal metastatic tumours	
N0/⩽5 *vs* >5	0 *vs* 1
	Total: 0–6
	
Tumor recurrence and death rates according to the PVN scores at 5 years from the initial operation	

**Classes (scores)**	**Cases**	**TRR (%)**	**MR (%)**
Low (0)	31	1 (3)	0
Int (1/2)	97	20 (21)	9 (9)
High (3)	42	23 (55)	19 (45)
Very high (4–6)	35	28 (80)	26 (74)
Total	205	72	54

PVN=primary tumour–vessel tumour–nodal tumour; IDC=invasive ductal carcinoma; absent=absent for event; TRR=tumour recurrence rate; MR=mortality rate; Int=intermediate; 1/2=1 and 2; 4–6=4 to 6.

**Table 3:** Crude disease-free and overall survival for PVN, pTNM, NPI, and HG classifications in patients with IDC without Nodal Metastasis and Positive for ER or PR or Both

	**PVN**				**PTNM**		
**Classes**	**Cases**	**TRR (%)**	**MR (%)**	**Stages**	**Cases**	**TRR (%)**	**MR (%)**
Low	74	4 (5)	1 (1)	I	66	5 (8)	2 (3)
Int	34	6 (18)	2 (6)	IIA	49	9 (18)	2 (4)
High	12	4 (33)	1 (20)	IIB	1	0	0
Very high	1	1 (100)	1 (100)	IIIB	5	1 (20)	1 (20)
Total	121	15	5	Total	121	15	5
							

**NPI**				**Modified NPI**			
**Classes**	**Cases**	**TRR (%)**	**MR (%)**	**Classes**	**Cases**	**TRR (%)**	**MR (%)**
Low	68	3 (4)	0	Low	68	3 (4)	0
Int	53	12 (23)	5 (9)	Int, low	29	8 (28)	2 (7)
High	0			Int, high	24	4 (17)	3 (13)
Total	121	15	5	High	0		
				Total	121	15	5

**HG**			
**Grades**	**Cases**	**TRR (%)**	**MR (%)**
1	39	2 (5)	0
2	48	7 (15)	0
3	34	6 (18)	5 (15)
Total	121	15	5
			

**Multivariate analyses**		
**Classifications**	**Disease-free survival**	
	**Trend HR/95% CI**	***P* for trend**
PVN	2.6/1.5–4.7	<0.001
pTNM	1.2/0.7–1.8	0.478
PVN	2.1/1.2–3.8	0.013
NPI	3.9/1.0–14.6	0.046
PVN	2.4/1.3–4.3	0.003
Modified NPI	1.6/0.8–3.1	0.150
PVN	2.5/1.4–4.4	0.002
HG	1.4/0.7–2.8	0.396

PVN=primary tumour–vessel tumour–nodal tumour; NPI=Nottingham Prognostic Index; HG=histologic grade; HR=hazard ratio; CI=confidence interval; TRR=tumour recurrence rate; MR=mortality rate.

Multivariate analyses were performed between PVN and pTNM, between PVN and NPI, and between PVN and HG.

**Table 4:** Crude disease-free and overall survival for PVN, pTNM, NPI, and HG classifications in patients with IDC without nodal metastasis and negative for both ER and PR

**PVN**				**pTNM**			
**Classes**	**Cases**	**TRR (%)**	**MR (%)**	**Stages**	**Cases**	**TRR (%)**	**MR (%)**
Low	36	2 (6)	2 (6)	I	35	4 (11)	4 (11)
Int	20	6 (30)	4 (20)	IIA	28	7 (25)	5 (18)
High	10	3 (30)	3 (30)	IIB	3	2 (67)	2 (67)
Very high	2	2 (100)	2 (100)	IIIB	2	0	0
Total	68	13	11	Total	68	13	11
							

**NPI**				**Modified NPI**			
**Classes**	**Cases**	**TRR (%)**	**MR (%)**	**Classes**	**Cases**	**TRR (%)**	**MR (%)**
Low	17	1 (6)	1 (6)	Low	17	1 (6)	1 (6)
Int	51	12 (24)	10 (20)	Int, low	25	4 (16)	4 (16)
High	0			Int, high	26	8 (31)	6 (23)
Total	68	13	11	High	0		
				Total	68	13	11

**HG**			
**Grades**	**Cases**	**TRR (%)**	**MR (%)**
1	6	0	0
2	18	2 (11)	2 (11)
3	44	11 (25)	9 (20)
Total	68	13	11
			

**Multivariate analyses**				
**Classifications**	**Disease-free survival**		**Overall survival**	
	**Trend HR/95% CI**	***P* for trend**	**Trend HR/95% CI**	***P* for trend**
PVN	2.7/1.4–5.0	0.002	2.7/1.4–5.0	0.002
pTNM	1.1/0.6–1.8	0.818	1.0/0.5–1.9	0.971
PVN	2.5/1.3–4.6	0.003	2.5/1.3–4.7	0.004
NPI	3.1/0.4–24.7	0.289	2.4/0.3–19.2	0.421
PVN	2.6/1.4–4.9	0.003	2.6/1.4–4.9	0.003
Modified NPI	2.0/0.9–4.8	0.110	1.6/0.7–3.9	0.299
PVN	2.8/1.5–5.2	0.001	2.8/1.5–5.3	0.001
HG	3.2/0.8–12.6	0.100	2.9/0.7–11.7	0.144

PVN=primary tumour–vessel tumour–nodal tumour; NPI=Nottingham Prognostic Index; HG=histologic grade; HR=hazard ratio; CI=confidence interval; TRR=tumour recurrence rate; MR=mortality rate.

Multivariate analyses were performed between PVN and pTNM, between PVN and NPI, and between PVN and HG.

**Table 5:** Crude disease-free and overall survival for PVN, pTNM, NPI, and HG classifications in patients with IDCs with nodal metastasis and positive for ER or PR or both

**PVN**				**PTNM**			
**Classes**	**Cases**	**TRR (%)**	**MR (%)**	**Stages**	**Cases**	**TRR (%)**	**MR (%)**
Low	23	2 (9)	0	IIA	26	0	0
Int	71	15 (21)	11 (15)	IIB	26	7 (27)	3 (12)
High	18	9 (50)	8 (44)	IIIA	30	10 (33)	8 (27)
Very high	18	13 (72)	12 (67)	IIIB	16	4 (25)	4 (25)
				IIIC	32	18 (56)	16 (50)
Total	130	39	31	Total	130	39	31
							

**NPI**				**Modified NPI**			
**Classes**	**Cases**	**TRR (%)**	**MR (%)**	**Classes**	**Cases**	**TRR (%)**	**MR (%)**
Low	6	0	0	Low	6	0	0
Int	54	9 (17)	5 (9)	Int, low	23	1 (4)	1 (4)
High	70	30 (43)	26 (37)	Int, High	31	8 (26)	4 (13)
Total	130	39	31	High	70	30 (43)	26 (37)
				Total	130	39	31
**HG**							

**Grades**	**Cases**	**TRR (%)**	**MR (%)**
1	20	5 (25)	3 (15)
2	66	15 (23)	12 (18)
3	44	19 (43)	16 (36)
Total	130	39	31
			

**Multivariate analyses**				
**Classifications**	**Disease-free survival**		**Overall survival**	
	**HR/95% CI**	***P*-value**	**HR/95% CI**	***P*-value**
PVN low	Referent		Referent	
Intermediate	2.0/0.4–8.9	0.380	Referent	
High	5.6/1.2–27.6	0.033	3.8/1.5–9.7	0.006
Very high	9.8/2.0–48.0	0.005	7.4/3.0–18.2	<0.001
	*P* for trend: <0.001		*P* for trend: <0.001	
				
pTNM IIA	Referent		Referent	
IIB	Referent		Referent	
IIIA	2.1/0.8–5.6	0.158	3.5/0.9–13.5	0.069
IIIB	1.1/0.3–4.1	0.837	2.6/0.6–12.2	0.215
IIIC	3.0/1.1–7.8	0.028	5.2/1.4–18.9	0.013
	*P* for trend: 0.019		*P* for trend: 0.020	
				
PVN low	Referent		Referent	
Intermediate	2.0/0.4–9.9	0.352	Referent	
High	5.3/1.0–28.1	0.048	3.4/1.3–9.1	0.012
Very high	11.8/2.2–64.4	0.005	8.1/3.1–21.1	<0.001
	*P* for trend: <0.001		*P* for trend: <0.001	
				
NPI low	Referent		Referent	
Intermediate	Referent		Referent	
High	1.6/0.7–3.9	0.289	2.4/0.8–7.2	0.109
	*P* for trend: 0.204		*P* for trend: 0.200	
				
PVN low	Referent		Referent	
Intermediate	1.3/0.3–6.2	0.757	Referent	
High	3.2/0.6–17.6	0.175	3.3/1.3–8.6	0.014
Very high	7.2/1.3–39.9	0.023	7.9/3.1–20.3	<0.001
	*P* for trend: <0.001		*P* for trend: <0.001	
				
Modified NPI low	Referent		Referent	
Intermediate, low	Referent		Referent	
Intermediate, high	7.1/0.8–59.0	0.071	3.4/0.4–30.7	0.271
High	7.7/0.9–65.1	0.061	5.7/0.7–45.5	0.100
	*P* for trend: 0.075		*P* for trend: 0.154	
				
PVN low	Referent		Referent	
Intermediate	3.3/0.7–15.0	0.120	Referent	
High	12.9/2.5–72.6	0.003	6.2/2.1–9.6	<0.001
Very high	32.9/6.0–182.2	<0.001	18.5/5.9–55.5	<0.001
	*P* for trend: <0.001		*P* for trend: <0.001	
				
HG 1	Referent		Referent	
2	0.5/0.2–1.6	0.274	0.8/0.2–3.0	0.718
3	0.4/0.1–1.3	0.129	0.5/0.1–2.2	0.375
	*P* for trend: 0.166		*P* for trend: 0.232	

PVN=primary tumour–vessel tumour–nodal tumour; NPI=Nottingham Prognostic Index; HG=histologic grade; HR=hazard ratio; CI=confidence interval; TRR=tumour recurrence rate; MR=mortality rate; Int=intermediate.

Multivariate analyses were performed between PVN and pTNM, between PVN and NPI, and between PVN and HG.

**Table 6:** Crude disease-free and overall survival for PVN, pTNM, NPI, and HG classifications in patients with IDCs with nodal metastasis and negative for both ER and PR

**PVN**				**pTNM**			
**Classes**	**Cases**	**TRR (%)**	**MR (%)**	**Stages**	**Cases**	**TRR (%)**	**MR (%)**
Low	8	0	0	IIA	16	4 (25)	4 (25)
Int	26	9 (35)	7 (27)	IIB	17	9 (53)	7 (41)
High	24	15 (63)	14 (58)	IIIA	14	6 (43)	5 (36)
Very high	17	15 (88)	15 (88)	IIIB	4	2 (50)	2 (50)
				IIIC	24	18 (75)	18 (75)
Total	75	39	36	Total	75	39	36
							

**NPI**				**Modified NPI**			
**Classes**	**Cases**	**TRR (%)**	**MR (%)**	**Classes**	**Cases**	**TRR (%)**	**MR (%)**
Low	1	0	0	Low	1	0	0
Int	27	8 (30)	6 (22)	Int, low	8	1 (13)	1 (13)
High	47	31 (66)	30 (64)	Int, high	19	7 (37)	5 (26)
Total	75	39	36	High	47	31 (66)	30 (64)
							
				Total	75	39	36

**HG**			
**Grades**	**Cases**	**TRR (%)**	**MR (%)**
1	3	0	0
2	29	11 (38)	9 (31)
3	43	28 (65)	27 (63)
Total	75	39	36
			

**Multivariate analyses**				
**Classifications**	**Disease-free survival**		**Overall survival**			
	**HR/95% CI**	***P*-value**	**HR/95% CI**	***P*-value**		
PVN low	Referent		Referent	
Intermediate	Referent		Referent	
High	3.5/1.4–8.7	0.007	3.9/1.4–10.8	0.009
Very high	9.1/3.3–25.3	<0.001	13.0/4.2–39.5	<0.001
	*P* for trend: <0.001		*P* for trend: <0.001	
				
pTNM IIA	Referent		Referent	
IIB	2.0/0.6–6.5	0.273	1.2/0.3–4.2	0.822
IIIA	0.9/0.2–3.5	0.896	0.6/0.1–2.4	0.458
IIIB	0.8/0.4–4.9	0.817	1.3/1.4–10.8	0.652
IIIC	1.4/0.4–4.9	0.569	1.3/0.4–4.7	0.652
	*P* for trend: 0.949		*P* for trend: 0.486	
				
PVN low	Referent		Referent	
Intermediate	Referent		Referent	
High	3.1/1.3–7.6	0.014	3.3/1.3–8.8	0.017
Very high	7.5/2.7–20.1	<0.001	17.6/3.4–29.3	<0.001
	*P* for trend: <0.001		*P* for trend: <0.001	
				
NPI low	Referent		Referent	
Intermediate	Referent		Referent	
High	1.4/0.6–3.5	0.488	1.7/0.6–4.6	0.324
	*P* for trend: 0.605		*P* for trend: 0.414	
				
PVN low	Referent		Referent	
Intermediate	Referent		Referent	
High	2.7/1.1–6.5	0.028	3.1/1.2–8.2	0.026
Very high	6.8/2.6–18.1	<0.001	9.4/3.2–27.6	<0.001
	*P* for trend: <0.001		*P* for trend: <0.001	
				
Modified NPI low	Referent		Referent	
Intermediate, low	Referent		Referent	
Intermediate, high	2.9/0.3–24.2	0.335	1.9/0.2–16.9	0.578
High	3.4/0.4–28.1	0.261	2.8/0.3–24.2	0.348
	*P* for trend: 0.453		*P* for trend: 0.403	
				
PVN low	Referent		Referent	
Intermediate	Referent		Referent	
High	3.2/1.4–7.4	0.008	3.6/1.4–9.2	0.007
Very high	7.5/2.9–18.6	<0.001	10.7/4.0–29.6	<0.001
	*P* for trend: <0.001		*P* for trend: <0.001	
				
HG 1	Referent		Referent	
2	Referent		Referent	
3	1.5/0.7–3.2	0.334	1.5/0.7–3.5	0.304
	*P* for trend: 0.394		*P* for trend: 0.359	

PVN=primary tumour–vessel tumour–nodal tumour; NPI=Nottingham Prognostic Index; HG=histologic grade; HR=hazard ratio; CI=confidence interval; TRR=tumour recurrence rate; MR=mortality rate; Int=intermediate.

Multivariate analyses were performed between PVN and pTNM, between PVN and NPI, and between PVN and HG.

## References

[bib1] Bloom HJG, Richardson WW (1957) Histological grading and prognosis in breast cancer. Br J Cancer 11: 359–577 13499785 10.1038/bjc.1957.43 PMC2073885

[bib2] Colpaert C, Vermeulen P, Jeuris W, van Beest P, Goovaerts G, Weyler J, Dam PV, Dirix L, Marck EV (2001) Early distant relapse in ‘node-negative’ breast cancer patients is not predicted by occult axillary lymph node metastases, but by the features of the primary tumour. J Pathol 193: 442–449 11276002 10.1002/path.829

[bib3] Cox DR (1972) Regression models and life-tables. J R Stat Soc 34: 187–220

[bib4] De Jong JS, Diest PJ van, Baak JPA (2000) Number of apoptotic cells as a prognostic marker in invasive breast cancer. Br J Cancer 82: 368–373 10646890 10.1054/bjoc.1999.0928 PMC2363300

[bib5] Depowski PL, Rosenthal SI, Ross JS (2001) Loss of expression of the PTEN gene protein product is associated with poor outcome in breast cancer. Mod Pathol 14: 672–676 11454999 10.1038/modpathol.3880371

[bib6] Donegan WL, Stine SB, Samter TG (1993) Implications of extracapsular nodal metastases for treatment and prognosis of breast cancer. Cancer 72: 778–782 8334631 10.1002/1097-0142(19930801)72:3<778::aid-cncr2820720324>3.0.co;2-j

[bib7] Elston CW, Ellis IO (1991) Pathological prognostic factors in breast cancer. I. The value of histological grade in breast cancer: experience from a large study with long-term follow-up. Histopathology 19: 403–410 1757079 10.1111/j.1365-2559.1991.tb00229.x

[bib8] Gilchrist KW, Gray R, Fowble B, Tormey DC, Taylor SG (1993) Tumor necrosis is a prognostic predictor for early recurrence and death in lymph node-positive breast cancer: a 10-year follow-up study of 728 eastern cooperative oncology group patients. J Clin Oncol 11: 1929–1935 8410120 10.1200/JCO.1993.11.10.1929

[bib9] Hasebe T, Mukai K, Tsuda H, Ochiai A (2000) New prognostic histological parameter of invasive ductal carcinoma of the breast: clinicopathological significance of fibrotic focus. Pathol Int 50: 263–272 10849311 10.1046/j.1440-1827.2000.01035.x

[bib10] Hasebe T, Sasaki S, Imoto S, Mukai K, Yokose T, Ochiai A (2002a) Prognostic significance of fibrotic focus in invasive ductal carcinoma of the breast: a prospective observational study. Mod Pathol 15: 502–516 12011255 10.1038/modpathol.3880555

[bib11] Hasebe T, Sasaki S, Imoto S, Ochiai A (2002b) Characteristics of tumors in lymph vessels play an important role in the tumor progression of invasive ductal carcinoma of the breast: a prospective study. Mod Pathol 15: 904–913 12218207 10.1097/01.MP.0000024289.59262.CE

[bib12] Hasebe T, Sasaki S, Imoto S, Ochiai A (2003a) Histological characteristics of tumors in blood vessels play an important role in tumor progression of invasive ductal carcinoma of the breast. Cancer Sci 94: 158–165 12708491 10.1111/j.1349-7006.2003.tb01413.x PMC11160227

[bib13] Hasebe T, Sasaki S, Imoto S, Ochiai A (2003b) Significance of nodal metastatic tumor characteristics in nodal metastasis and prognosis of patients with invasive ductal carcinoma of the breast. Cancer Sci 94: 181–187 12708494 10.1111/j.1349-7006.2003.tb01416.x PMC11160216

[bib14] Hasebe T, Sasaki S, Imoto S, Ochiai A (2004a) Prognostic significance of the intra-vessel tumor characteristics of invasive ductal carcinoma of the breast: a prospective study. Virchows Arch 444: 20–27 14624360 10.1007/s00428-003-0921-0

[bib15] Hasebe T, Sasaki S, Imoto S, Ochiai A (2004b) Histological characteristics of tumors in vessels and lymph nodes are significant parameter for predicting tumor progression of invasive ductal carcinoma of the breast: a prospective study. Hum Pathol 35: 298–308 15017585 10.1016/j.humpath.2003.05.004

[bib16] Kaplan EL, Meier P (1958) Nonparametric estimation from incomplete observations. J Am Stat Assoc 53: 457–481

[bib17] Pedersen AN, Christensen IJ, Stephens RW, Briand P, Mouridsen HT, Dano K, Brunner N (2000) The complex between urokinase and its type-1 inhibitor in primary breast cancer relation to survival. Cancer Res 15: 6927–6934 11156392

[bib18] Scorilas A, Karameris A, Arnogiannaki N, Ardavanis A, Bassilopoulos P, Trangas T, Talieri M (2001) Overexpression of matrix-metalloproteinase-9 in human breast cancer. A potential favourable indicator in node-negative patients. Br J Cancer 84: 1488–1496 11384099 10.1054/bjoc.2001.1810 PMC2363667

[bib19] Sobin LH, Wittekind CH. TNM Classification of Malignant Tumors 6th edn. New York (NY): Wiley-Liss, 2002 pp 131–141

[bib20] Sundquist M, Thorstenson S, Brudin L, Nordenskjold B, the South East Swedish Breast Cancer Study Group (1999) Applying the Nottingham Prognostic Index to a Swedish breast cancer population. Breast Cancer Res Treat 53: 1–8 10206067 10.1023/a:1006052115874

[bib21] Todd JH, Dowle C, Williams MR, Elston CW, Ellios IO, Hinton CP, Blamey RW, Haybittle JL (1987) Confirmation of a prognostic index in primary breast cancer. Br J Cancer 56: 489–492 3689666 10.1038/bjc.1987.230 PMC2001834

